# Comparison of *Helicobacter pylori* stool antigen, salivary IgG, serum IgG, and serum IgM as diagnostic markers of *H. pylori* infection in children

**Published:** 2019-06

**Authors:** Andy Darma, Bagus Samsu Tri Nugroho, Vinny Yoanna, Indah Sulistyani, Alpha Fardah Athiyyah, Reza Gunadi Ranuh, Subijanto Marto Sudarmo

**Affiliations:** Department of Child Health, Dr. Soetomo Hospital, Faculty of Medicine Universitas Airlangga, Surabaya, Indonesia

**Keywords:** *Helicobacter pylori*, Health professional shortage areas (HpSA), Salivary IgG, Serum IgG and IgM, Endoscopy-biopsy

## Abstract

**Background and Objectives:**

Various non-invasive diagnostic tests are available for the detection of *Helicobacter pylori* infection. The aim of this study was to compare the sensitivity and specificity of HpSA, salivary IgG, serum IgG, and serum IgM to those of endoscopic-biopsy as the gold standard for the diagnosis of *H. pylori* infection.

**Materials and Methods:**

This is a cross-sectional study performed among pediatric patients at Dr. Soetomo General Hospital (Surabaya, Indonesia). Fecal, blood, and saliva samples were collected from all subjects. The results of the HpSA, salivary IgG, serum IgG, and serum IgM tests were compared to the results of endoscopic-biopsy as the gold standard.

**Results:**

Of the 37 study participants, *H. pylori* infection was confirmed in 5 (13.33%) with serum IgG, 23 (63.33%) with serum IgM, 15 (40%) with HpSA, and 26 (70.97%) with salivary IgG. The salivary IgG enzyme-linked immunosorbent assay (ELISA) was the only diagnostic test with significantly different results, as compared to biopsy (p = 0.017).

**Conclusion:**

The results of this study showed that HpSA, salivary IgG, and serum IgG and IgM were not sufficient to replace endoscopic-biopsy as the gold standard for the diagnosis of *H. pylori* infection.

## INTRODUCTION

*Helicobacter pylori* is an important bacterial agent that mediates various gastrointestinal diseases ranging from gastritis to gastric cancer. Although previously unexpected to survive in the low pH of the stomach (1–3), *H. pylori* has been found to play a significant role in the development of peptic ulcers (4, 5). In children, *H. pylori* infection is considered significant and can lead to various gastrointestinal problems, such as pediatric halitosis (6), peptic ulcer (7), repeated vomiting, iron malabsorption (8, 9), and chronic gastritis (10). Although up to one-third of world’s children population are reportedly infected with *H. pylori*, infected children are often asymptomatic (11, 12). In contrast, only 3.8% of children in Indonesia were found to be infected with *H. pylori* (13). This phenomenon emphasizes the need for a sensitive diagnostic method.

The guidelines of the European Society for Paediatric Gastroenterology Hepatology and Nutrition (ESPGHAN) and North American Society for Pediatric Gastroenterology, Hepatology and Nutrition (NASPGHAN) state that endoscopic biopsy is an important component for the initial detection of *H. pylori* infection (14). However, this method is invasive, high-risk, expensive, uncomfortable to the patient, and requires a specially-skilled operator (15). The stool antigen test and urea-breath test are considered more accurate than serological antibody-based tests for the detection of *H. pylori* infection (16, 17). While the urea-breath test has sensitivity of 88%–95% and specificity of 95%–100%, it is relatively expensive and may expose the operator to radioactivity (18).

There are also non-conventional methods to detect *H. pylori*. For example, *H. pylori* stool antigen (HpSA) immunochromatography has been used to detect the microorganism in fecal samples (19). An enzyme-linked immunosorbent assay (ELISA) has also been developed for the detection of *H. pylori* in saliva (20) and serum (21) samples. Unlike endoscopy and the urea-breath test, which are observer-based assessments, these techniques rely on laboratory tools to detect the microorganism. However, comparative data between these non-conventional methods and endoscopic biopsy are lacking. Therefore, the aim of the present study was to compare the sensitivity and specificity of HpSA, salivary immunoglobulin (Ig) G, serum IgG, and IgM, to those of endoscopic-biopsy as the gold standard for the diagnosis of *H. pylori* infection.

## MATERIALS AND METHODS

### Study design.

In this study, the sensitivity, specificity, negative predictive value, positive predictive value, positive likelihood ratio (PLR), and negative likelihood ratio (NLR) of HpSA, salivary IgG, serum IgG, and IgM were compared to those of endoscopic-biopsy for the detection of *H. pylori* infection. The study protocol was approved by the ethics Committee of Dr. Soetomo Hospital (approval no. 03/Panke. KKE/I/2012).

### Population and samples.

The study cohort was comprised of pediatric patients who visited Dr. Soetomo Hospital (Surabaya, Indonesia) from May to July of 2012. Samples were collected in the outpatient clinic and pediatric ward. The inclusion criteria were as follows: age of 3–18 years, clinical signs of *H. pylori* infection (i.e., at least three episodes abdominal pain over the last 3 months), symptoms of dyspepsia (i.e., repeated episodes of epigastric pain, abdominal discomfort, bloating, nausea, vomiting, early satiety, and post-meal abdominal distention within the last 3 months with initial onset at 6 months before complaints), and willingness of parents or guardians to consent to research participation. Exclusion criteria were as follows: previous administration of antibiotics, H2-antagonists, or proton-pump inhibitors for 4 weeks prior to examinations, and evidence of co-infection. Biopsy for diagnostic assessment was performed and fecal samples were collected from subjects who fulfilled the inclusion criteria.

### Endoscopic-biopsy, specimen collection and examination.

Sample collection was performed in the Internal Medicine Endoscopy Room of Dr. Soetomo General Hospital by experts who were blinded to the aims of this study. Biopsy specimens were obtained via endoscopy, then fixated with 10% formaldehyde solution and stained with hematoxylin/eosin and malachite green, and viewed under a high-power field with a light microscope. Anatomical pathologists were assigned to assess each sample. Finally, the findings of *H. pylori* in biopsy specimens were reported as positive or negative.

### Fecal specimen collection and examination.

At least 5 g of feces were collected in conventional fecal containers and stored within 1 h. All samples were temporarily stored at 2°C for a maximum of 72 h. Samples from patients with diarrhea that were exposed to room temperature for more than 4 h were rejected. Immunochromatographic HpSA was assessed using a rapid stool antigen test (ACON Laboratories, Inc., San Diego, USA) by the Prodia Laboratory (Diponegoro, Surabaya, Indonesia). For HpSA examination, 1–2 g of feces were collected and diluted in buffer. Two strips were used to verify a positive result, while one strip was used to confirm a negative result.

### Blood specimen collection and examination.

Blood samples were collected by Prodia Laboratory and centrifuged at 5000 rpm for 5 min to obtain supernatant, which was then sent for immediate immunochromatographic analysis of serum IgM and IgG using specific ELISAs (Helicolisa IgM: PT Indec Diagnostics East Jakarta, Indonesia; Euroimmun IgG: Euroimmun, Lübeck, Germany). An optical density of 20 RU/mL (relative units per mL) was used as minimum positive cutoff for IgG, while the positive cutoff for IgM was > 0.90 ID U (index units).

### Collection and examination of saliva samples.

After fasting for at least 60 min, each patient was instructed to gargle and spit into a small container. Salivary specimens were collected by Prodia Laboratory. All collected specimens were immediately sent for salivary IgG examination. Specimens that required storage were refrigerated at −20°C to −8°C. Salivary IgG concentrations were measured using an immunochromatographic ELISA with peroxidase-conjugated anti-human IgG as an enzyme conjugate (Monobind Inc., Lake Forest, CA, USA). An optical density of 20 U/mL was the minimum positive cutoff for salivary IgG detection.

## RESULTS

In total, 37 randomly selected children (54.1% males) participated in this study. The most frequent complaints were abdominal pain (94.6%) and vomiting (70.3%) (Table 1). *H. pylori* infections were detected through biopsy, anti-*H. pylori* serum IgG, anti-*H. pylori* serum IgM, HpSA, and salivary IgG. As shown in Table 2, salivary IgG had the highest positive results of *H. pylori* infection in children besides biopsy, followed by anti-*H. pylori* serum IgM, while anti-*H. pylori* serum IgG had highest negative results.

**Table 1.** Characteristics of study participants

	**N**	**%**
**Age (months)**		
36–71	9	24.32
72–95	3	8.11
96–119	11	29.73
≥120	14	37.84
**Sex**		
Male	20	54.05
Female	17	45.95
**Complaints**		
Abdominal pain	35	94.59
Nausea	21	56.76
Vomiting	26	70.27
Early satiety	5	13.51
**Family History of GERD**		
Yes	14	37.84
No	23	62.16

**Table 2.** Comparison of *H. pylori* diagnostic test results (Biopsy, Serum IgM and IgG, HpSA, Salivary IgG-ELISA)

**Diagnostic Test**	**N**	**%**
**Biopsy**		
Positive	31	83.78
Negative	6	16.22
**Serum IgG**		
Positive	4	13.33
Negative	26	86.67
**Serum IgM**		
Positive	19	63.33
Negative	11	36.67
**HpSA**		
Positive	12	40.00
Negative	18	60.00
**Salivary IgG**		
Positive	22	70.97
Negative	9	29.03

Overall, the results of this study showed that the diagnostic tests had high sensitivities, but poor specificities. The anti-*H. pylori* IgG ELISA was the most sensitive test for the diagnosis of *H. pylori* infection in children, while HpSA was the least sensitive. Although the IgG ELISA was the most sensitive, it was the least specific (Table 3).

**Table 3.** Diagnostic values of serum IgM and IgG, HpSA, salivary IgG-ELISA

**Diagnostic Test**	**Diagnostic Value**						***p***

	**Sensitivity**	**Specificity**	**PPV**	**NPV**	**PLR**	**NLR**	
Serum IgG	100.00%	15.38%	15.38%	100.00%	1.18	0.18	1.000
Serum IgM	94.74%	27.27%	69.23%	75.00%	1.30	0.89	0.126
HpSA	91.67%	22.22%	44.00%	80.00%	1.18	0.40	0.622
Salivary IgG	95.45%	44.44%	80.77%	80.00%	1.72	2.31	0.017*

PPV: positive predictive value; NPV: negative predictive value; PLR: positive likelihood ratio; NLR: negative likelihood ratio; *p* value based on result comparison with biopsy using chi-square

The chi-square test was used to compare the positivity rates among the diagnostic tests with the biopsy results. In this study, the salivary Ig-G ELISA was the only diagnostic test with significantly different results, as compared to biopsy (*p* = 0.017).

## DISCUSSION

At present, upper endoscopy with biopsy is the gold standard for the diagnosis of *H. pylori* infection (15). For an initial diagnosis of *H. pylori* infection, the ESPGHAN and NASPGHAN guidelines recommend to use the invasive gastric biopsy-based methods, such as (1) positive bacterial culture and (2) *H. pylori* gastritis, as confirmed by histopathological analysis with a positive result of at least one other test (rapid urease test, PCR, or fluorescence *in situ* hybridization), but no non-invasive tests (14).

HpSA is a non-invasive diagnostic tool. A study conducted in Turkey reported that HpSA had a sensitivity of 98% and specificity of 100% (22). False-negative results with the HpSA test might be caused by low-intensity *H. pylori* colonization and subsequent lower fecal concentration in children (23). False-positive results for HpSA detection could arise from interspecies cross-reactions of antibodies with the use of an HpSA rapid strip and other urease producting-*Helicobacter* species, such as *H. pullorum* and *H. canis*. However, a previous study failed to show that the HpSA assay would give false-positive results with other *Helicobacter* species (24), indicating the need for further studies to confirm such cross-reactions among *Helicobacter* species.

Salivary IgG is a simple and comfortable method for the diagnosis of *H. pylori* infection and does not increase the risk of infection due to venous puncture. A study conducted in England reported that salivary IgG had a sensitivity of 94% and specificity of 85% (25). The results of the present study showed that the salivary IgG test is useful for screening rather than diagnosis. However, the statistically significant difference between the salivary IgG test and biopsy results showed the need for further studies to evaluate the reliability of these tests. A false-positive result of the salivary IgG test can result from *H. pylori*-related periodontal disease or actively bleeding gums (26), which could explain the sensitivity and specificity of salivary IgG in this study, since poor dental health was not included as an exclusion criterion. On the other hand, a false-negative result can occur due to the low IgG concentration in children that is below the detection limit of the ELISA. Besides, the best method for saliva collection is by passively allowing the saliva to flow through the lower lips (27, 28), but this method is difficult for smaller children.

Serum IgG and IgM ELISAs should be considerable as options because these methods are readily available, easy, practical, and cheap, and testing accuracy is not affected by ulcer bleeding, gastric atrophy, or the use of proton pump inhibitors or antibiotics (29). However, this test is not reliable for the evaluation of eradication therapy, since antibody levels can persist for extended periods (29). A previous study reported that sensitivity and specificity of serum IgG were 87.6% and 61.0%, respectively, while those of serum IgM for patients aged ≤17 years were 9.0% and 97.0%, respectively (21). Different *H. pylori* strains existing in different geographical areas may result in undetected strains due to the high specificities of immunoglobulins (30). The difference between the cut off values of serum IgM-IgG level in an adult population versus children can also result in false-negative results (31). The low specificity in this study also resulted from immunological cross reactions among different bacteria other than *H. pylori* that can stimulate antibody production, similar to that produced by *H. pylori* (30).

The PLR reflects the proportion of true-positive results of sick subjects and false-positive results of normal subjects. In contrast, the NLR reflects the proportion of false-negative results of sick subjects and true-positive results of normal subjects (32). Specificity and sensitivity play significant roles in determining the NLR. Hence, the NLR is useful to investigate the probability of a diagnostic test to yield a deviant result. In the present study, it appears that all of the alternative diagnostic tests had a moderate PLR, as compared to biopsy, demonstrating that a high probability of *H. pylori* detection can be expected with the use of alternative diagnostic tests. However, the high NLR of most of the alternative diagnostic tests, with the exception of serum IgG, indicates that the test results of truly infected and normal subjects did not differ much. Salivary IgG had the lowest negative predictive value at 2.31, meaning that there is a higher chance of a negative result of infected subjects.

In this study, the salivary IgG results significantly differed from the biopsy results. On the contrary, a diagnostic approach using serum IgM, serum IgG, or HpSA appears to yield results similar to those of biopsy, which is reflected by the high sensitivity of the evaluated tests. However, the specificities were low for serum IgM, serum IgG, and HpSA, which is in accordance with the guidelines of the ESPGHAN and NASPGHAN, stating the unnecessity of antibody testing for diagnosis of *H. pylori* infection (14). Therefore, the causes of false-negative and false-positive results should be further investigated.

The results of the present study showed that serum IgM, serum IgG, and HpSA are not sufficient for the diagnosis of *H. pylori* infection. Nonetheless, regardless of specificity, serum IgM, serum IgG, and HpSA are sufficiently sensitive for screening of *H. pylori* infection (33). Thus, it appears that serum IgM, serum IgG, and HpSA may be useful for non-invasive screening of *H. pylori* infection in children, rather than as diagnostic tests.

## CONCLUSION

Current tests for the diagnosis of *H. pylori* infection in children are challenging for pediatricians. While different guidelines are available, choices of diagnostic modalities for *H. pylori* detection are limited. The results of this study demonstrated that serum IgM, serum IgG, HpSA and salivary IgG failed to meet the criteria of a good diagnostic test to replace currently recommended invasive and non-invasive diagnostic methods for the detection of *H. pylori*.

Further studies are needed to confirm our findings. Study limitations, such as the low number of samples and patient refusal of sample collection, may have affected the study results. Moreover, only one reagent was used in this study to measure each respective variable, thus it may be difficult to compare to reagents from different manufacturers. Investigations using different ELISA reagents based on different *H. pylori* strains may be needed to confirm these differences.
